# Safety and Efficacy of Atogepant for Migraine Prevention: A Systematic Review and Meta-Analysis of Randomized Controlled Trials

**DOI:** 10.7759/cureus.112994

**Published:** 2026-07-20

**Authors:** Samahir Akram Nizamani, Ramsha Memon, Chinenye Iguh, Roa Ali, Onuwabhagbe Daniel Omomhenle, Nathaniel Oluwakayode Oyedeji, Sadia Zafar, Austin Nwawueze, Hurmat Amjad, Paul Sunday Samuel, Daud Gul

**Affiliations:** 1 Neurology, Liaquat University Hospital, Jamshoro, PAK; 2 Medicine, Windsor University School of Medicine, Cayon, KNA; 3 Neurology, Royal Victoria Infirmary, Newcastle upon Tyne, GBR; 4 Psychiatry, University of Benin, Benin City, NGA; 5 Psychiatry, Tees, Esk and Wear Valleys NHS Foundation Trust, Middlesbrough, GBR; 6 Psychiatry, Ladoke Akintola University of Technology, Ogbomoso, NGA; 7 Neurology, Sahiwal Medical College, Sahiwal, Sahiwal, PAK; 8 Psychiatry, University of Calabar, Calabar, NGA; 9 Medicine, Fatima Jinnah Medical University, Lahore, PAK; 10 Medicine, Harrogate and District NHS Foundation Trust, Harrogate, GBR; 11 Geriatrics, Abia State University, Uturu, NGA; 12 Geriatrics, Barking, Havering and Redbridge University Hospitals NHS Trust, London, GBR; 13 Medicine, Bridgeport Medical Clinic, Waterloo, CAN

**Keywords:** atogepant, cgrp receptor antagonist, chronic migraine, efficacy, episodic migraine, meta-analysis, migraine prevention, randomized controlled trials, safety

## Abstract

Migraine represents a widespread neurological disorder that causes substantial disability and impacts on quality of life and functioning. An oral calcitonin gene-related peptide (CGRP) receptor antagonist, atogepant, is a new targeted preventive treatment for migraine. This systematic review and meta-analysis aimed to evaluate the effectiveness and safety profile of atogepant in migraine prevention. According to the PRISMA guidelines, relevant studies were identified through a thorough database search in the PubMed, Cochrane, and ScienceDirect databases to identify all randomized controlled trials (RCTs) evaluating atogepant in adults with episodic or chronic migraine. A total of six high-quality RCTs and 3,453 patients were included. The outcomes evaluated were monthly migraine days (MMDs), monthly headache days (MHDs), acute migraine medication use, and adverse events. Pooled analysis demonstrated that atogepant significantly reduced MMDs (mean difference (MD) = -1.47; 95% CI -1.73 to -1.21), MHDs (MD = -1.71; 95% CI -1.95 to -1.48), and acute migraine medication use (MD = -1.69; 95% CI -1.94 to -1.44) compared with placebo across dose levels. Higher doses with reductions were observed, particularly with the 60 mg once-daily and 30 mg twice-daily doses. Atogepant was effective in hard-to-treat populations such as those with chronic migraine and headaches related to medication overuse. The overall risk of adverse events was slightly higher with atogepant than with placebo (RR = 1.13; 95% CI 1.01 to 1.25), although most events were mild to moderate, including nausea, constipation, fatigue, and decreased appetite. Findings from the subgroup analyses indicated that the 30 mg twice-daily regimen may be linked to an increased risk of adverse events, suggesting differences in tolerability between doses. In general, atogepant was well tolerated and safe, demonstrating good safety and tolerability relative to other traditional preventive migraine medications. This study provides evidence supporting the effectiveness and tolerability of atogepant as an oral preventive treatment for episodic and chronic migraine.

## Introduction and background

Migraine is a frequent and disabling neurological condition involving periodic attacks of headache with associated symptoms of nausea, photophobia, and phonophobia [1]. It has a substantial impact on quality of life (QoL) and significantly increases the global burden of disability and health care [2]. It affects more than one billion people worldwide and remains one of the leading causes of years lived with disability globally [2]. Preventive treatment of migraine is available, but numerous conventional preventive agents have poor adherence and adverse effect profiles that could affect the efficacy of preventive treatment [3,4]. In the last few years, improved insight into migraine mechanisms has highlighted calcitonin gene-related peptide (CGRP) as a major contributor to migraine pathogenesis, and, as a consequence, to the emergence of targeted, CGRP-based migraine prevention drugs [5].

Atogepant is a new oral CGRP receptor antagonist specifically designed to treat migraine prevention [6]. Recent migraine management guidelines recognize CGRP-targeted therapies as effective preventive treatment options and recommend considering them as first-line therapies for migraine prevention [7]. In contrast to other preventive agents, atogepant directly inhibits the CGRP pathway and offers the benefits of oral administration, rapid therapeutic activity, and better tolerability [8]. Early dose-finding studies evaluated both once-daily (QD) and twice-daily (BID) dosing to characterize the efficacy, safety, and tolerability of different dosing regimens. The findings from these studies informed the selection of dosing regimens for subsequent phase III trials in patients with episodic and chronic migraine [8,9]. Several randomized controlled trials (RCTs) have shown that atogepant can decrease the frequency of monthly migraine days (MMDs) and improve patient outcomes, with generally mild-to-moderate side effects [10,11]. But the differences in dosing schedules, patient populations, and clinical outcomes reported in various trials have led to inconsistencies in its overall safety and efficacy profile [10,11]. Moreover, the majority of the previous reviews focused on the entire category of CGRP-targeted drugs instead of prioritizing atogepant [12,13].

This study synthesizes evidence on atogepant, a novel oral CGRP receptor antagonist for migraine prevention, from recently published RCTs. Unlike previous systematic reviews that primarily evaluated CGRP-targeted therapies as a class, the present review focuses specifically on atogepant. It provides a comprehensive, dose-stratified evaluation in patients with episodic and chronic migraine. By incorporating the latest clinical evidence, this review offers an updated assessment of atogepant's efficacy, safety, and tolerability, providing clinically relevant guidance for migraine prevention.

This study aims to assess the safety and efficacy of atogepant for migraine prevention by summarizing the evidence from RCTs. More specifically, the purpose of this study is to evaluate the efficacy of atogepant in reducing the frequency of migraine attacks, the drug's safety (adverse events related to the drug and its tolerability), and to compare its effectiveness against placebo or other therapies.

## Review

Methods

We conducted this systematic review and meta-analysis to assess the efficacy and safety of atopegant for migraine prevention, following PRISMA guidelines [14]. The review protocol was also registered on PROSPERO (CRD420261399058).

Search Strategy

Our search strategy used keywords combined with Boolean operators. We used three main databases: Cochrane, PubMed, and ScienceDirect. The search strategy we used for PubMed was ‘((((atogepant) OR (calcitonin gene-related peptide receptor antagonist)) OR (MK-8031)) AND (Migraine)) OR (chronic migraine)’ and for Cochrane we used ‘(atogepant): ti, ab,kw OR (calcitonin gene-related peptide receptor antagonist): ti, ab,kw OR (MK-8031): ti, ab,kw AND (Migraine): ti, ab,kw OR (chronic migraine): ti, ab,kw’. For ScienceDirect, we used ‘(((atogepant) OR (calcitonin gene-related peptide receptor antagonist) OR (MK-8031)) AND ((Migraine) OR (chronic migraine))). We restricted our search to articles published after 2000. For PubMed and Cochrane, we used trial filters, and for ScienceDirect, we used a research article filter. Additionally, we searched the reference lists of the included articles for any relevant articles.

Selection Criteria and Outcomes

We included studies of patients diagnosed with migraine according to the criteria of the International Classification of Headache Disorders, aged 18 years or older, who received atogepant as an intervention, irrespective of dose; the study design needed to be an RCT to be included. Additionally, we only included studies reporting on our safety and efficacy outcomes.

Articles were excluded if they were not RCTs or if they were RCTs that were not published. We excluded studies using atogepant in combination with some other migraine medication or not using atogepant as the main intervention. Studies were excluded if they were not in English or if they were conference abstracts. Studies not reporting on any of the included outcomes or providing outcomes in any non-measurable format were excluded.

We assessed efficacy using mean MMDs, mean monthly headache days (MHDs), and mean monthly acute migraine medication use. We assessed safety using the occurrence of adverse events.

Screening, Selection, and Data Extraction

We retrieved articles from our included databases into EndNote (Clarivate, London, UK) to search for duplicates using the duplicate checker and manually removed any remaining duplicates. We searched the remaining articles using their titles and abstracts for relevance and removed all that were irrelevant at this stage. We searched the full text of the remaining articles and screened those with full text available against our eligibility criteria. Articles fulfilling our criteria were included in the final analysis. The whole process was carried out independently by two reviewers, who then screened it. External help was sought where required, and any conflict regarding the inclusion of an article was resolved with the help of another reviewer.

Data extraction was carried out by one reviewer and reviewed by another. The process consisted of extracting data into an Excel sheet (Microsoft Corp., Redmond, WA, USA). We extracted author names, study year, design, location, migraine type, intervention details, outcomes, sample size, and age.

Quality Assessment and Data Synthesis

We assessed the quality of the included RCTs using the Risk of Bias 2 (RoB 2) tool [15]. The studies were regarded as high risk, low risk, or of some concern. Studies were considered high risk if one domain was high risk or more than one domain had some concerns. Studies were classified as having some concerns if one domain had some concerns, while the remaining domains were low risk. Studies were considered low risk if all domains had a low risk of bias.

RevMan 5.4 (The Cochrane Collaboration, London, England, UK) was used to analyze the data from included studies. For continuous variables, we used mean differences (MDs) as the outcome measure, and for dichotomous variables, we used risk ratios with inverse-variance weighting and the Mantel-Haenszel method. We used a random-effects model for all outcomes and reported 95% confidence intervals. Any outcome with a p-value below 0.05 was considered significant. Statistical heterogeneity was measured using I^2^; only values above 40% were considered significant enough to explore. I^2^ below 25% was considered low, mild for 26-50%, moderate for 51-75%, and high for >75%. A leave-one-out sensitivity analysis and subgroup analysis were conducted where possible. Publication bias could not be assessed using a funnel plot due to the limited data available.

Results

**Figure 1:**
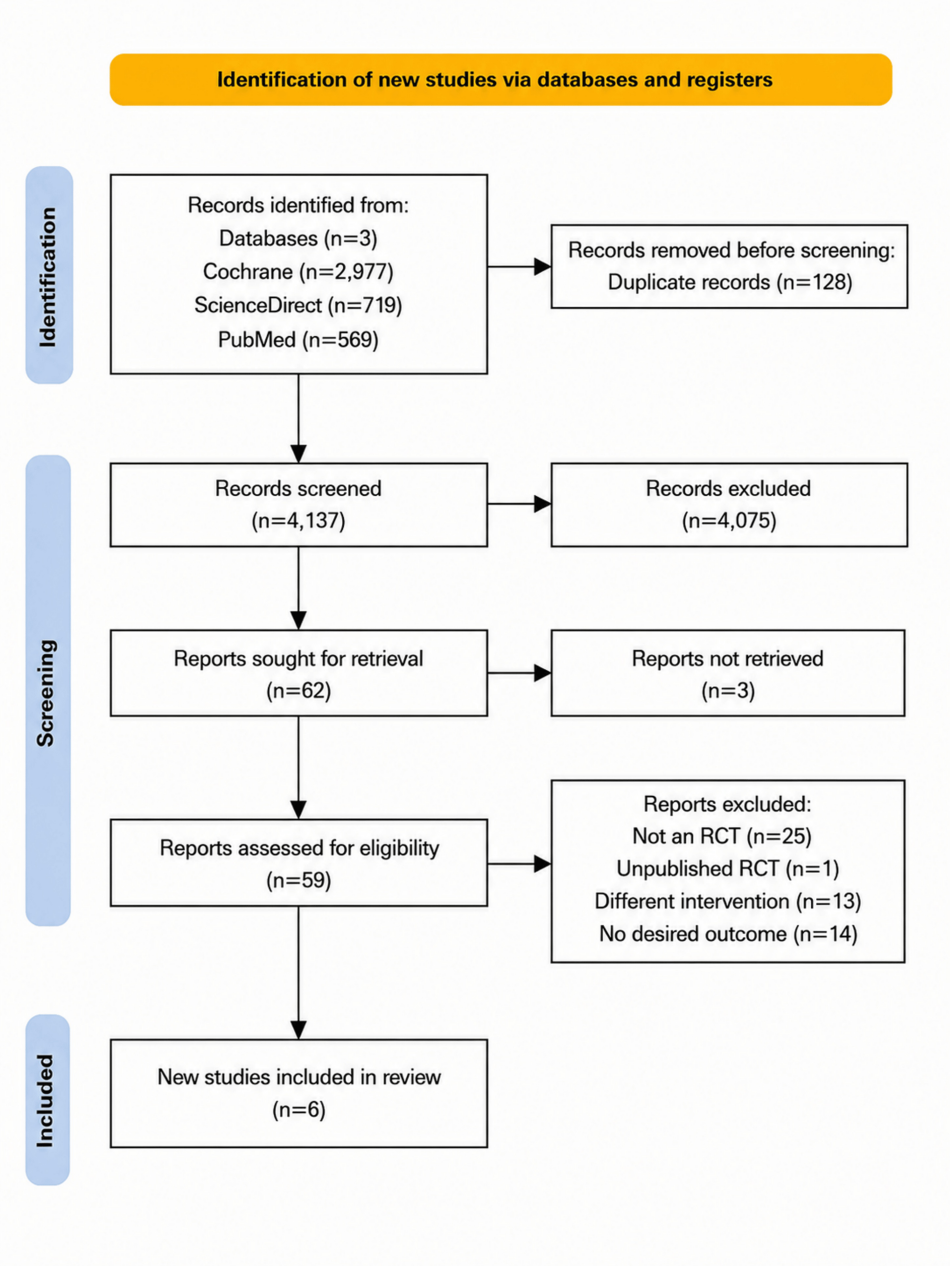
PRISMA flowchart for study screening and selection RCT: randomized controlled trial

We initially retrieved 4265 articles with 569 from PubMed, 2977 from Cochrane, and 719 from ScienceDirect. A total of 128 duplicates were identified and removed. Title and abstract screening were performed on the remaining articles, and 4,075 articles were excluded for being irrelevant. The full text of three articles could not be found; thus, 59 articles were screened against our eligibility criteria. Only six articles could fulfill our selection criteria and were included in the final analysis [10,16-20]. The screening and selection process is summarized in Figure 1.

Characteristics of Studies

Six randomized, double-masked, placebo-controlled trials were included, totaling 3,453 participants. The studies, which were published between 2020 and 2025, included a specific Japanese cohort as well as foreign settings. The pooled sample was mostly female (83-89%), with mean ages ranging from 38.5 to 44.8 years. Four trials included individuals with episodic migraine (4-14 migraine days/month), whereas two focused on chronic migraine (≥15 headache days/month). Over 12 weeks, all studies compared oral atogepant at varied doses (10, 30, or 60 mg QD; 30 or 60 mg BID) with placebo. The primary goal of all trials was a change from baseline in mean MMDs, with secondary outcomes including headache days, acute medication use, responder rates, and quality-of-life indicators. All of the included trials were high quality, with low risk of bias according to the ROB2 assessment (Table 1).

**Table 1:** Characteristics of included studies QD: once daily; BID: twice daily; M: male; F: female; MMDs: monthly migraine days; MHDs: monthly headache days; MSQ: Migraine-Specific Quality of Life Questionnaire; AIM-D: Activity Impairment in Migraine-Diary; HIT-6: Headache Impact Test-6; MIDAS: Migraine Disability Assessment; PGIC: Patient Global Impression of Change; PHQ-9: Patient Health Questionnaire-9; PDA: Performance of Daily Activities; PI: Physical Impairment; ROB 2: Revised Cochrane Risk of Bias Tool; SD: standard deviation All included studies were randomized, double-blind, placebo-controlled trials and were assessed as having a low risk of bias.

Author	Sample Size	Age, Mean (SD)	Sex (M/F)	Objective	Intervention	Population Diagnosis	Outcomes Assessed	ROB2 Result
Goadsby PJ et al., 2020 [19]	Placebo: n = 178; Atogepant 10 mg QD: n = 92; Atogepant 30 mg QD: n = 182; Atogepant 60 mg QD: n = 177; Atogepant 30 mg BID: n = 79; Atogepant 60 mg BID: n = 87	Placebo: 40.5 (11.7) 10 mg QD: 39.4 (12.4) 30 mg QD: 41.0 (13.6) 60 mg QD: 40.4 (11.7) 30 mg BID: 38.5 (11.2) 60 mg BID: 39.7 (11.9)	M: 111 (13%); F: 714 (87%)	Evaluate the safety, tolerability, and efficacy of oral atogepant doses for episodic migraine prevention	Placebo vs atogepant 10/30/60 mg QD or 30/60 mg BID × 12 weeks	Episodic migraine 4-14 migraine days/month ≥1-year history	Primary: Change from baseline in mean MMDs; Secondary: MHDs; ≥50% responder rate; acute medication use days	Low risk
Ailani J et al., 2021 [10]	Placebo: n = 214; Atogepant 10 mg QD: n = 214; Atogepant 30 mg QD: n = 223; Atogepant 60 mg QD: n = 222	Placebo: 40.3 (12.8) 10 mg QD: 41.4 (12.0) 30 mg QD: 42.1 (11.7) 60 mg QD: 42.5 (12.4)	M: 101 (11.2%); F: 801 (88.8%)	Confirm efficacy and safety of atogepant QD for episodic migraine prevention	Placebo vs atogepant 10/30/60 mg QD × 12 weeks	Episodic migraine 4-14 migraine days/month Onset <50 years	Primary: Change from baseline in mean MMDs; Secondary: MHDs; acute medication use days; ≥50% responder rate; MSQ Role Function-Restrictive; AIM-D Performance of Daily Activities; AIM-D Physical Impairment	Low risk
Goadsby PJ et al., 2024 [20]	With overuse: Placebo: n = 169; Atogepant 30 mg BID: n = 161; Atogepant 60 mg QD: n = 170; Without overuse: Placebo: n = 77; Atogepant 30 mg BID: n = 92; Atogepant 60 mg QD: n = 86	Placebo: 42.0 (12.4) 30 mg BID: 42.6 (11.9) 60 mg QD: 41.7 (12.3)	M: 96 (12.4%); F: 659 (87.6%)	Evaluate atogepant efficacy in chronic migraine with/without acute medication overuse.	Placebo vs atogepant 30 mg BID or 60 mg QD × 12 weeks; subgroup by medication overuse status	Chronic migraine: ≥15 headache days/month, ≥8 migraine days/month, 66.2% with acute medication overuse	Primary: Change from baseline in mean MMDs; Secondary: MHDs; acute medication use days; ≥50% responder rate; HIT-6; MSQ (RFR, RFP, EF); AIM-D (PDA, PI); MIDAS; PGIC; PHQ-9; proportion meeting medication overuse criteria	Low risk
Matsumori Y et al., 2025 [18]	Placebo: n = 133; Atogepant 10 mg QD: n = 127; Atogepant 30 mg QD: n = 130; Atogepant 60 mg QD: n = 131	Placebo: 44.8 (10.5) 10 mg QD: 41.3 (11.7) 30 mg QD: 43.2 (11.4) 60 mg QD: 42.5 (10.3)	M: 86 (16.4%); F: 437 (83.6%)	Evaluate atogepant efficacy and safety in Japanese episodic migraine patients	Placebo vs atogepant 10/30/60 mg QD × 12-week DBTP + 12-week active extension	Episodic migraine, 4-14 migraine days/month, Japanese population (100%), Onset <50 years	Primary: Change from baseline in mean MMDs; Secondary: MHDs; acute medication use days; ≥50% responder rate; MSQ v2.1 RFR domain; AIM-D PDA and PI domain scores	Low risk
Tassorelli C et al., 2024 [17]	Placebo: n = 157; Atogepant 60 mg QD: n = 156	Placebo: 43.4 (10.3) 60 mg QD: 40.9 (10.7)	M: 33 (11%); F: 280 (89%)	Evaluate the safety, tolerability, and efficacy of oral atogepant 60 mg QD for preventive treatment of episodic migraine in patients for whom two to four conventional oral treatment classes failed.	Placebo vs atogepant 60 mg QD × 12 weeks	Episodic migraine; history of two to four conventional oral preventive treatment failures; Adults (18-80 years)	Primary: Change from baseline in mean MMDs across 12 weeks; Secondary: Safety, tolerability, adverse event profile (e.g., constipation), and treatment discontinuation rates	Low risk
Pozo-Rosich P et al., 2023 [16]	Placebo: n = 259; Atogepant 60 mg QD: n = 262; Atogepant 30 mg BID: n = 257	Total safety population: Mean: 42.1 years (SD not explicitly detailed in abstract text)	M: 96 (12%); F: 677 (88%)	Evaluate the efficacy, safety, and tolerability of atogepant for the preventive treatment of chronic migraine.	Placebo vs atogepant 60 mg QD or 30 mg BID × 12 weeks	Chronic migraine: Adults aged 18-74 years with a one-year or longer history of chronic migraine	Primary: Change from baseline in mean MMDs across 12 weeks; Secondary: Safety, tolerability, specific adverse events (constipation, nausea), and weight decrease metrics	Low risk

**Figure 2:**
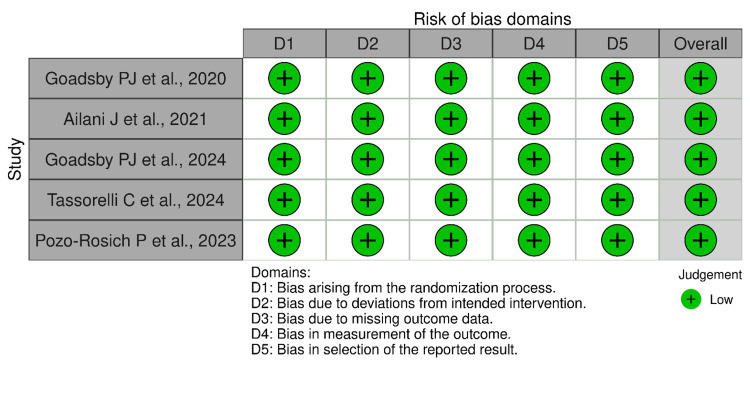
Summary of risk of bias Studies included [19,10,20,17,16]

The summary of the ROB is given in Figure 2.

Effect of MMDs

**Figure 3:**
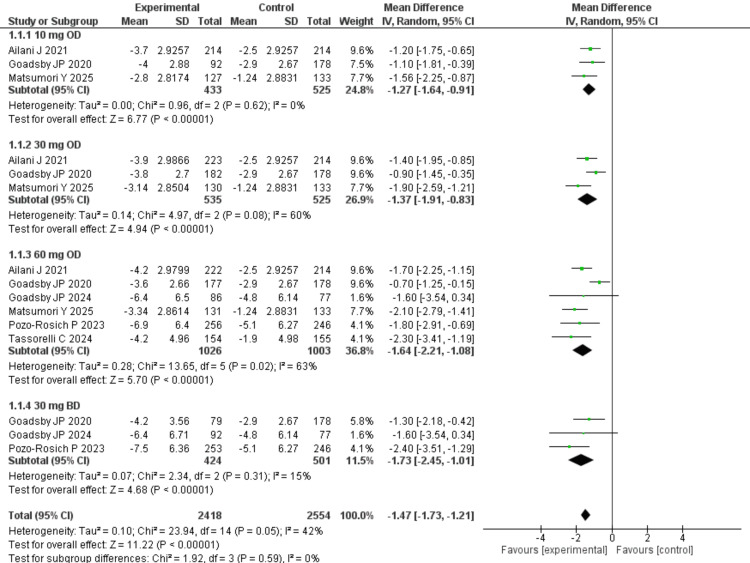
Forest plot showing results of MMDs Studies included [19,10,20,18,17,16] MMDs: monthly migraine days; OD: once daily; BID: twice daily

Overall, there was a significantly reduced mean MMDs in the atogepant group compared to placebo (MD = -1.47; 95% CI = -1.73, -1.21; p < 0.00001), with mild heterogeneity (42%). We conducted a subgroup analysis by dose. In the 10 mg OD group, the number of MMDs was significantly lower than in the placebo group, with no heterogeneity (MD = -1.27; 95% CI = -1.64, -0.91, p < 0.00001). In the 30 mg OD group, a significantly reduced MMD was observed, with moderate heterogeneity (I^2^ = 60%; MD = -1.37; 95% CI = -1.91, -0.83; p < 0.00001). In the 60 mg OD group, similar to other groups, a significantly lower number of MMDs was observed with moderate heterogeneity of 63% (MD -1.64; 95% CI -2.21, -1.08, p < 0.00001), and results were significantly in favor of 30 mg BD group as well and heterogeneity was low (MD -1.73; 95% CI -2.45, -1.01, p < 0.00001). The results are shown in Figure 3.

Sensitivity analysis showed that removing Goadsby JP 2020 [19] reduced heterogeneity to 0% in the 60 mg OD group, whereas results remained unchanged when other studies were removed.

Effect on MHDs

**Figure 4:**
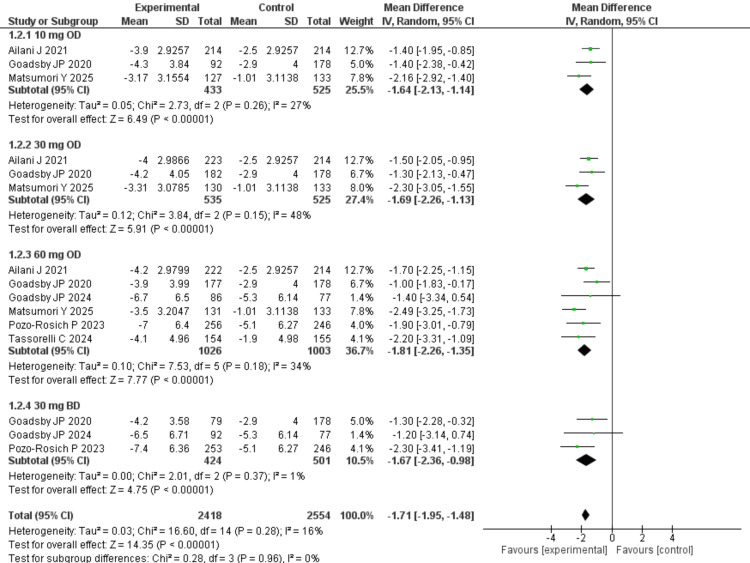
Forest plot showing results of MHDs Studies included [19,10,20,18,17,16] MHDs: monthly headache days; OD: once daily; BID: twice daily

The analysis showed that the atogepant group had a significantly lower mean monthly headache-day count than the placebo group (MD = -1.71; 95% CI = -1.95, -1.48; p < 0.00001), with low heterogeneity (16%). We did dose-based subgroup analyses. The 10 mg OD group had a considerably lower number of MHDs than the placebo group, with mild heterogeneity (27%; MD -1.64; 95% CI -2.13, -1.14, p < 0.00001). In the 30 mg OD group, MHDs were considerably lower, but there was mild heterogeneity (I^2^ = 48%) (MD -1.69; 95% CI -2.26, -1.13, p < 0.00001). The 60 mg OD group, like the other groups, had a significantly reduced number of MHDs with mild heterogeneity of 34% (MD -1.81; 95% CI -2.26, -1.35, p < 0.00001), and the results were also significantly in favor of the 30 mg BD group (MD -1.67; 95% CI -2.36, -0.98, p < 0.00001), which had low heterogeneity of 1%. The results are displayed in Figure 4.

Effect on Medication Use

**Figure 5:**
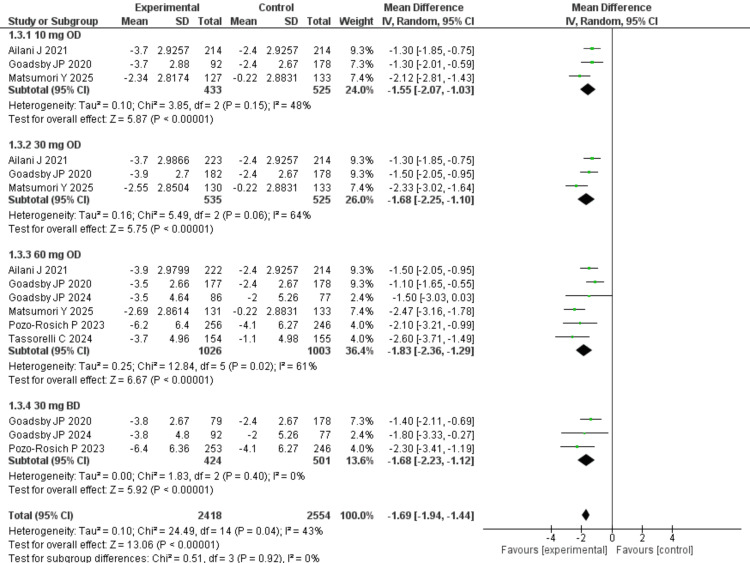
Forest plot showing results of acute medication use Studies included [19,10,20,18,17,16] OD: once daily; BID: twice daily

The analysis of acute medication use revealed that the atogepant group had substantially less acute medication use than the placebo group (MD = -1.69; 95% CI = -1.94, -1.44; p < 0.00001), with mild heterogeneity (43%). We conducted dose-based subgroup analyses. The 10 mg OD group had significantly fewer medication uses than the placebo group (MD = -1.55; 95% CI = -2.07, -1.03; p < 0.00001), with mild heterogeneity of 48%. Acute medication use in the 30 mg OD group was significantly lower (MD = -1.68; 95% CI = -2.25, -1.10, p < 0.00001); however, there was moderate heterogeneity (I^2^ = 64%). The 60 mg OD group, like the other groups, had a much lower number of medications uses, with moderate heterogeneity of 61% (MD -1.83; 95% CI -2.36, -1.29, p < 0.00001), and the findings were likewise significantly in favor of the 30 mg BD group (MD -1.68; 95% CI -2.23, -1.12, p < 0.00001), which had no heterogeneity (Figure 5).

A leave-one-out sensitivity analysis showed that heterogeneity became insignificant (i.e., <40%) upon removing Goadsby JP 2020 [19].

Adverse Events

**Figure 6:**
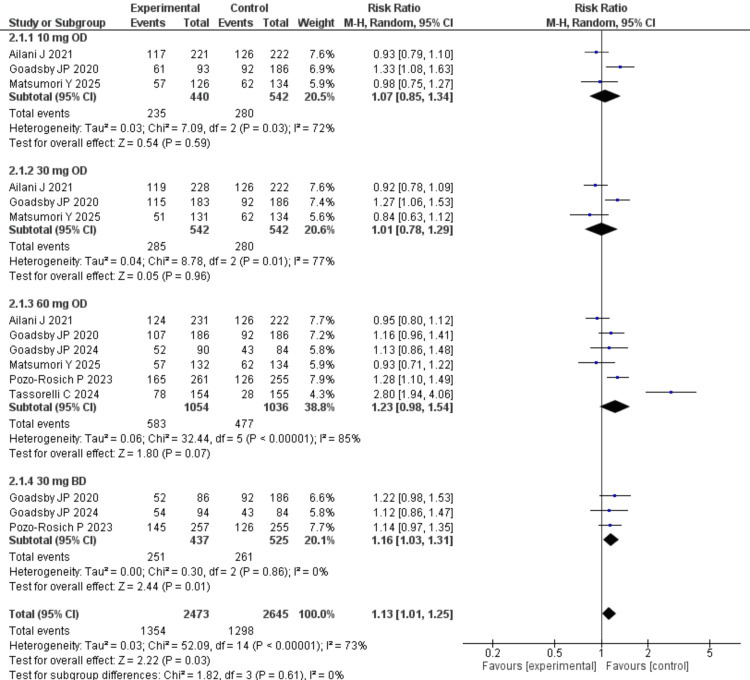
Forest plot showing risk of adverse events Studies included [19,10,20,18,17,16] OD: once daily; BID: twice daily

We also observed adverse events to assess the safety of atogepant. Our analysis showed that the risk of adverse events was higher with atogepant overall, but moderate heterogeneity of 73% was observed (RR 1.13; 95% CI 1.01, 1.25, p = 0.03). Upon subgroup analysis, we found no significant difference in the risk of adverse events between the 10 mg OD atogepant group and the placebo group, with moderate heterogeneity (72%; RR 1.07; 95% CI 0.85, 1.34; p = 0.59). No significant difference could be observed between the 30 mg OD (RR 1.01; 95% CI 0.78, 1.29, p = 0.96) and 60 mg OD groups (RR 1.23; 95% CI 0.98, 1.54, p = 0.07), with high heterogeneity in both groups. However, the risk of adverse events was significantly higher in the 30 mg BD group than in the placebo group (RR 1.16; 95% CI 1.03, 1.31, p = 0.01) (Figure 6).

Although heterogeneity remained high, removal of Ailani J 2021 [10] during the sensitivity analysis of the 60 mg OD group significantly favored placebo over atogepant.

Discussion

This study examined the safety and efficacy of atogepant for migraine prevention, drawing on six high-quality RCTs involving 3,453 participants. The results showed that atogepant was effective in reducing MMDs, MHDs, and acute migraine medication use compared with placebo across various doses. These results support the growing evidence that CGRP receptor antagonism is an effective therapeutic strategy for migraine prevention and further establish atogepant as a promising oral preventive treatment option for both episodic and chronic migraine. The results are consistent with all previous RCTs and meta-analyses, which also showed reductions in MMD and MHD, as well as reduced acute medication use, compared to placebo in people who received atogepant, and an improved safety and tolerability profile [11,21].

We observed a significant decrease across all atogepant dose groups, and the decrease in MMDs appeared to be greater with higher doses, including 60 mg once daily and 30 mg twice daily. The results are consistent with the biological function of CGRP in migraine pathophysiology, in which blocking the CGRP receptor has been shown to decrease trigeminovascular activation and neurogenic inflammation [10,22]. The observed reduction in MHDs and acute medication use further supports the clinical benefits of treatment with atogepant beyond reducing migraine frequency; as fewer patients rely on acute medications, fewer people may experience a negative impact on QoL and a reduced risk of developing medication-overuse headache (MOH).

An important strength of this meta-analysis is that it included a heterogeneous group of patients with migraine, including episodic migraine, chronic migraine, patients with medication overuse, and a Japanese cohort, which enhanced the generalizability of the results. The PROGRESS and ELEVATE trials showed that atogepant continued to be effective in populations of difficult-to-treat patients with prior treatment failure, several preventive treatment options, and in those with chronic migraine with medication overuse [16,17]. This study indicates that atogepant may be clinically relevant and used as a targeted migraine-specific preventive treatment in patients with limited treatment choices.

In terms of safety, atogepant demonstrated a favorable tolerability profile, although a modest increase in adverse events was observed in the pooled analysis. The most common adverse events reported in the trials included mild-to-moderate symptoms, such as nausea, constipation, fatigue, and decreased appetite. The results of this study are consistent with previous pooled safety analyses and meta-analyses, which reported a similarly favorable tolerability profile for atogepant, with most treatment-emergent adverse events mild to moderate and treatment discontinuation rates low [23,24]. Subgroup analysis demonstrated that adverse event risk was more pronounced with the 30 mg twice-daily regimen, suggesting possible dose-related tolerability differences, consistent with previous studies reporting increased treatment-emergent adverse events with higher atogepant doses [22,23]. Nevertheless, the overall safety profile remained favorable compared with many traditional migraine preventive medications, which are often associated with poor adherence due to intolerable side effects.

Variability in efficacy and safety outcomes may be attributed to differences in study design, patient populations, migraine subtype, baseline migraine frequency, treatment duration, and dosing regimens across the included trials. Sensitivity analyses further supported the robustness of the findings. Removal of the Goadsby et al. (2020) study substantially reduced heterogeneity in the analyses of MMDs and acute migraine medication use. This may be explained by its phase 2b/3 dose-ranging design, which evaluated multiple QD and BID regimens, unlike the subsequent confirmatory phase III trials. In contrast, exclusion of the Ailani et al. (2021) study altered the significance of the 60 mg QD adverse event analysis, likely because this large confirmatory phase III trial contributed substantially to the pooled safety estimates through its larger sample size and standardized study design. Nevertheless, the overall direction of treatment effects remained consistent across sensitivity analyses, supporting the robustness and reliability of the primary findings.

Limitations

The present study has some limitations to note when interpreting the findings. First, only six RCTs were included, and they were not suitable for detailed subgroup analyses and publication bias assessment. Although the review used a comprehensive search method, the possibility of publication bias cannot be ignored. Secondly, differences in dosing regimens, migraine subtype, baseline disease severity, and patient populations led to some heterogeneity in several outcomes. Third, the length of follow-up in the majority of the included trials was relatively brief, typically 12 weeks or fewer, limiting assessment of the interventions' long-term efficacy, safety, and adherence. Fourth, the proportion of females among the participants was high, which may limit the applicability of the results to males. Last, the included studies were mainly head-to-head trials between atogepant and placebo, and trials directly head-to-head with other CGRP-targeted agents or traditional preventive medications are limited. Furthermore, as only English-language publications were included, the possibility of language bias cannot be excluded.

Recommendations

Large-scale, long-term randomized trials across different patient populations to evaluate the long-term efficacy and safety of atogepant are indicated. Further comparative studies between atogepant and other CGRP antagonists, monoclonal antibodies, and conventional preventive treatments are required to understand atogepant's clinical effectiveness better. Patient-reported outcomes, cost-effectiveness, treatment adherence, and effectiveness in real-world clinical settings should also be investigated in additional studies. In addition, studies on optimal dosing and predictors of treatment response could help personalize migraine prevention therapy and enhance patient outcomes.

## Conclusions

This study demonstrated that atogepant is an effective and safe option for migraine prophylaxis. Atogepant was effective in decreasing MMDs, MHDs, and acute medication use compared to placebo across several dose regimens. While a slight increase in the occurrence of adverse events was noted, most were mild to moderate. The results confirm the potential of atogepant as a targeted oral preventive treatment for both episodic and chronic migraine and its effectiveness as a new tool to enhance and optimize migraine treatment in patients with a suboptimal response to current treatment.
